# T-Lymphocyte Subsets in the Embryonic Spleen Undergoing a Graft-Versus-Host Reaction

**DOI:** 10.1155/1991/37107

**Published:** 1991

**Authors:** Barbara Fedecka-Bruner, Pierre Vaigot, Jacqueline Désveaux-Chabrol, Marcelle Gendreau, Guido Kroemer, Françoise Dieterlen-Lièvre

**Affiliations:** 1 Institut d'Embryologie du CNRS et du Collge de France 49bis Avenue de la Belle Gabrielle F-94736 Nogent-sur-Marne Paris Cedex France; 2 Centro de Biologia Molecular CSIC Universidad Autonoma Madrid 28049 Spain

## Abstract

Allogeneic immunocompetent T cells injected into chicken embryos induce a graft-versushost reaction (GVHR) whose most prominent manifestation is splenic hyperplasia. The highly inbred CC and CB strains of chickens used here are, respectively, homozygous for the B4 or B12 MHC haplotypes. By means of a panel of immunological reagents, including alloantisera and monoclonal antibodies against public domains of the T-cell receptor, CD4, CD8, and the inducible interleukin-2-receptor light chain (CD25), it is shown that the bulk of cells in the enlarged spleen are of host origin and do not express markers typical of mature T or B lymphocytes. Among recipient splenocytes, the quantitatively most important population consists of TCRαβ^-^TCRγδ^-^CD4^-^CD8^+^CD25^+^ (TCR0) lymphocytes. Donor cells encountered in the spleen prevalently exhibit a TCRαβ^+^CD4^+^CD8^-^CD25^+^ phenotype and proliferate in vivo. The data demonstrate that nonspecific host and potentially specific donorderived cellular elements contribute to splenomegaly.


*Developmental Immunology*, 1991, Vol. 1, pp. 163–168
Reprints available directly from the publisher
Photocopying permitted by license only

© 1991 Harwood Academic Publishers GmbH
Printed in the United Kingdom

### T-Lymphocyte Subsets in the Embryonic Spleen Undergoing a Graft-Versus-Host Reaction

BARBARA FEDECKA-BRUNER*†, PIERRE VAIGOT†, JACQUELINE DÉSVEAUX-CHABROL†, MARCELLE GENDREAU†, GUIDO KROEMER §, and FRANÇOISE DIETERLEN-LIÈVRE†

*†Institut d'Embryologie du CNRS et du Collège de France, 49bis Avenue de la Belle Gabrielle, F-94736 Nogent-sur-Marne, Paris Cedex, France*

Allogeneic immunocompetent T cells injected into chicken embryos induce a graft-versus-host reaction (GVHR) whose most prominent manifestation is splenic hyperplasia. The highly inbred CC and CB strains of chickens used here are, respectively, homozygous for the B4 or B12 MHC haplotypes. By means of a panel of immunological reagents, including alloantisera and monoclonal antibodies against public domains of the T-cell receptor, CD4, CD8, and the inducible interleukin-2-receptor light chain (CD25), it is shown that the bulk of cells in the enlarged spleen are of host origin and do not express markers typical of mature T or B lymphocytes. Among recipient splenocytes, the quantitatively most important population consists of $TCR\alpha\beta^-TCR\gamma\delta^-$ $CD4^-CD8^+CD25^+$ (TCR0) lymphocytes. Donor cells encountered in the spleen prevalently exhibit a $TCR\alpha\beta^+CD4^+CD8^-CD25^+$ phenotype and proliferate *in vivo*. The data demonstrate that nonspecific host and potentially specific donor-derived cellular elements contribute to splenomegaly.

KEYWORDS: Graft-versus-host reaction, lymphocyte subsets, chicken embryo.

### INTRODUCTION

The development of graft-versus-host reactions (GVHR) depends on a special set of requirements each of which has the status of *conditio sine qua non* (for a review, see Bril and Benner, 1985; Parkman, 1989). First, graft and host have to differ in major or minor histocompatibility loci. Second, the allograft must contain immunologically competent alloreactive T cells. Third, the recipient needs to be immunodeficient, thus being incapable of rapidly eliminating the grafted cells by an immunological counterattack (host-versus-graft reaction). GVHR is a major complication of therapeutic bone marrow transplantation in patients and may become manifest in an acute or chronic fashion, then mimicking certain aspects of autoimmune diseases. Much of the pioneering work on GVHR has been performed in the chicken embryo, where it is induced either by placing immunocompetent allogeneic T cells onto the chorioallantoic membrane or by injecting them intravenously (Simonsen, 1957, 1962; Seto and Albright, 1965; Désveaux-Chabrol and Dieterlen-Lièvre, 1987; Désveaux-Chabrol et al., 1989). The latter procedure entails a systemic response whose most spectacular manifestation is splenomegaly, that is, an easily quantifiable increase of splenic mass.

Extensive studies have been carried out on the T-cell subsets required to trigger the reaction in the mammalian adult GVHR model (Gleichman et al., 1984; Korngold and Sprent, 1985; Sprent et al., 1990). In this model, the GVHR usually develops in an irradiated animal, whose own responding cells have been largely eradicated. On the other hand, in the avian embryonic model, host cells proliferate actively especially in the early phase of the reaction, as shown earlier using sex chromosomes as markers (Owen et al., 1965; Seto and Albright, 1965; McBride, 1966; Nisbet and Simonsen, 1967). Precocious immunological maturation of the host immune system in serial transfer experiments was hypothesized to occur under the influence of allogeneic cells (McBride et al., 1966; Lafferty et al., 1972; Simonsen, 1985).

*Corresponding author.
§Present address: Centro de Biología Molecular, CSIC, Universidad Autonoma, Campus de Cantoblanco, 28049 Madrid, Spain.

In these studies however, subpopulations of host and donor cells were not investigated for the want of specific markers, so that the enlarged spleen remained largely a black box. We recently showed by means of a pan T-cell monoclonal antibody (mAb) (Yassine et al., 1989) that the T-cell population became amplified by a factor of 10 or more in the spleens of embryos undergoing a GVHR (Fedecka-Bruner et al., 1989). For the present study, we have used two inbred chicken strains, carrying, respectively, the B4 and B12 MHC haplotypes, and have performed double staining of spleen cells, to combine detection of MHC haplotype and one of several functional markers.

Panels of different monoclonal antibodies (mAbs) specific for chicken cluster of differentiation (CD) antigens have become available, allowing the study of the expression of clonotypic (TCR1, equivalent of mammalian $\gamma/\delta$TCR, Sowder et al., 1988; TCR2 = $\alpha/\beta$ TCR, Chen et al., 1988) or invariant (CD3, Chen et al., 1986) components of the CD3/TCR complex, as well as the distribution of CD4, CD8 (Chan et al., 1988), and IL-2-receptor (IL-2R) light chains (CD25, Mr = 50 kD, Hàla et al., 1986; Schauenstein et al., 1988). Using these mAbs in conjunction with alloantisera, we evaluated the relative contribution of donor and recipient in an attempt to determine to which extent specific and nonspecific phenomena are involved in GVHR-induced splenomegaly.

### RESULTS

### Relative Contribution of Donor and Host Cells to Splenomegaly

As depicted in Fig. 1, the numeric contribution of lymphoid cells increases in an overproportional fashion during GVHR-induced splenomegaly (Figs. 1A and 1B). Among these lymphoid elements, a significant portion exhibits allogeneic markers of the donor MHC haplotype ($B^4$) and appears to vigorously proliferate *in vivo*, increasing by a factor of 5.1 from E18 to 20 of embryonic age and reaching up to $6.5 \times 10^6$ cells (Fig. 1C). Given that probably only a fraction of the injected PBL ($10^6$ cells) homes to the recipient's spleen, the increase of donor cells probably is more important than suggested by these numbers. Nonetheless, the majority of cells (approximately 90% at E18 and 80% at E20) present in the hyperplastic spleen (about 10 times control weight at E18 to E20) is of host origin. Thus, *in situ* proliferation of recipient cells in the enlarging spleen and/or recruitment of cells from other organs quantitatively outnumber donor cells.

### Characterization of T-Lymphocyte Subpopulations

Splenomegaly produced in the ($B^4$) donor–($B^{12}$) recipient combination is accompanied by an increase of $CD8^+$ cells (from about $2 \times 10^4$ in controls to $5 \times 10^6$

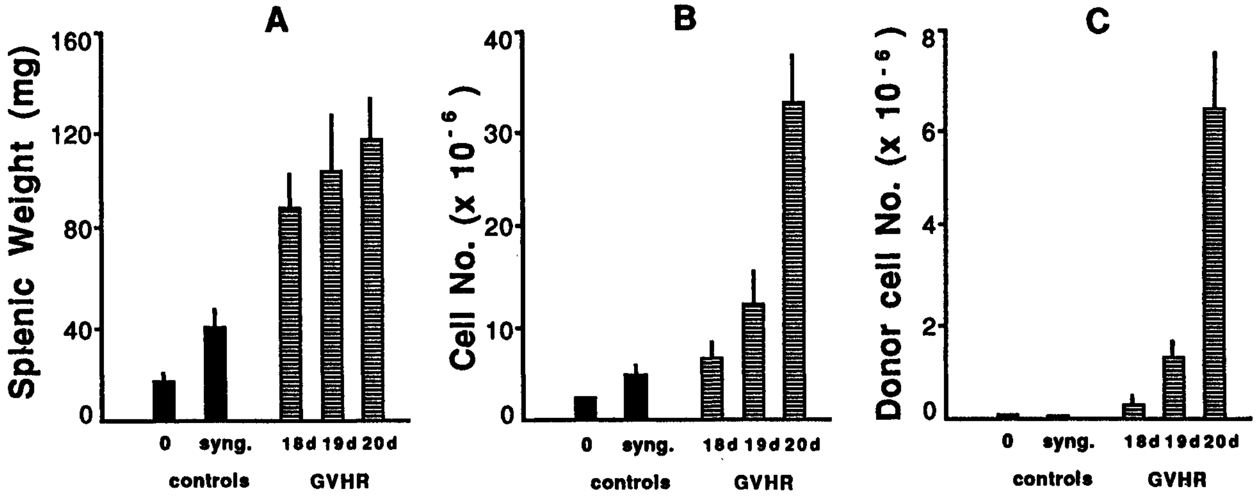

FIGURE 1. Lymphoid cell numbers and donor-cell contribution in GVHR-induced splenomegaly. One million $B^4$ or $B^{12}$ lymphocytes (3- to 4-week-old donors) were injected i.v. into E13 old $B^{12}$ embryos. At E18, E19, or E20 of embryonic age, splenic weight (A), total leukocyte numbers (B), and the number of cells reacting with a $B^4$)-specific alloantiserum (C) were determined. $86 \pm 2\%$ of cells isolated from spleens undergoing GVHR on E18 to E20 were stained by a $B^{12}$-specific alloantiserum. Only E20 untreated (0) or syngeneic controls (syng.) are shown; these control values do not significantly change from E18 to E20. Note the overproportional increase in lymphoid cells and cells of the donor phenotype. Values were determined by microscope counting for (B) and by microscope counting and cell-sorter analysis for (C).

on E20 in embryos undergoing GVHR), as well as by the appearance of cells that are practically absent in control spleens, namely, lymphoid cells exhibiting CD4, TCR1, TCR2, IL-2R, or a B-cell marker (Fig. 2). From E18 to 20, the percentage of the $CD8^+$ cells among splenocytes increases significantly, whereas the percentages of other cell populations remain relatively constant (although their absolute number increases, Fig. 1). Nonetheless, the majority of splenocytes isolated from chicken embryos undergoing GVHR lack surface antigens normally found on lymphocytes. Thus, nonlymphoid or prelymphoid (i.e., nonspecific) elements are important in the generation of splenic hyperplasia. Double-staining experiments reveal that $CD4^+$ cells are predominantly of the donor and $CD8^+$ cells of the recipient MHC haplotype (Table 1). Though double staining for CD4 or CD8 and TCR1 or TCR2 was not performed, it is possible to infer from the values in Table 1 that most $CD4^+$ cells (mainly of donor origin) express TCR2. On the other hand, Fig. 2 shows that cells displaying TCR1 are scarce. It follows that most $CD8^+$ cells, the bulk of which originates from the recipient embryo, carry neither TCR1 nor TCR2. These $CD8^+$ cells thus probably correspond to the cells found by Bucy et al. (1989, 1990) ($CD4^-CD8^{dull+}TCR1^-TCR2^-TCR3^-$/surface $CD3^-$ cytoplasmic $CD3^+$) that may represent the avian equivalent of mammalian natural killer cells (so-called TCR0 cells) and that develop in the unmanipulated embryonic spleen independently of the thymus.

### Lymphocyte Subsets Expressing Interleukin-2-Receptor Light Chains (CD25)

In untreated or syngeneic controls, CD25 is faintly expressed only on a small ($<2\%$) subpopulation of embryonic spleen cells. In contrast, up to 15% of cells strongly express CD25 in spleens of chicks undergoing GVHR. The majority of these $CD25^+$ cells bear the Class I MHC recipient phenotype (Table 1). Most $CD8^+$ cells (84%) and a large portion of $CD4^+$ cells (65%) express CD25 on E20, that is, 7 days after injection of allogeneic adult PBL (Table 1). These findings strongly suggest that IL-2-mediated proliferation and/or differentiation are implicated in the expansion of these cell subsets.

### CONCLUDING REMARKS

The data shown in this paper suggest the following scenario of GVHR-induced splenomegaly. After intravenous injection, a portion of donor lymphocytes home to the embryonic spleen, where they are

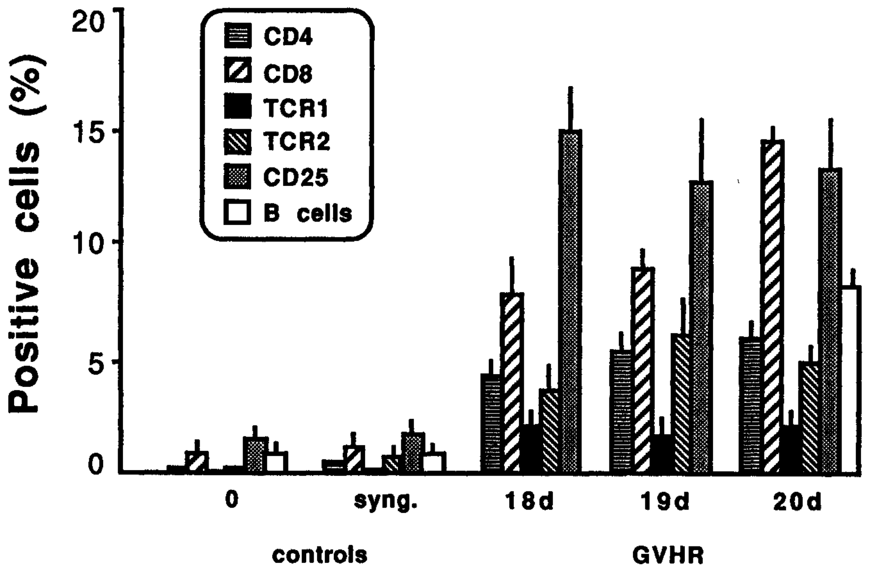

FIGURE 2. Lymphocyte subsets in GVHR-induced splenomegaly. Splenocytes (same animals as in Fig. 1) were subjected to immunofluorescence analysis using a panel of monoclonal antibodies. No significant difference was found in lymphoid cell number and lymphocyte subset distribution between E18 and E20 syngeneic controls (E20 values shown). The percentage of B cells (open bars) were only determined for E20. Cell counts were determined by microscope examination and Facs analysis.

TABLE 1
Assignation of Lymphocyte Subsets to the Donor ($B^4$) or host ($B^{12}$) MHC Haplotype and Characterization of Cells Expressing the IL2-R Light Chain (CD25)[a]

| Subpopulation | Percentage of cells positive among respective subpopulations for | | |
|---|---|---|---|
| | B4 | B12 | CD25 |
| All lymphoid cells | 19.6 ± 0.2 | 77.4 ± 3.5 | 13.2 ± 1.1 |
| CD4 | 91.7 ± 0.8 | 5.6 ± 0.4 | 66.8 ± 5.0 |
| CD8 | 22.5 ± 0.5 | 76.5 ± 3.5 | 83.9 ± 8.2 |
| TCR1 | ND[b] | ND | 100 |
| TCR2 | 87.5 ± 2.5 | 14.0 ± 2.1 | 71.1 ± 13.6 |
| CD25 | 22 ± 6 | 77 ± 7 | 100 |
| $B^4$ | 100 | — | 37.5 ± 5.8 |
| $B^{12}$ | — | 100 | 62.5 ± 6.4 |

[a]IF double staining followed by microscope counting and cell-sorter analysis were applied to splenocytes from E20 embryos undergoing a GVHR.
[b]ND = not determined.

activated and start proliferating in response to allogeneic antigens, mainly MHC determinants, once they become expressed during ontogeny. Because the expression of Class II MHC antigen precedes that of Class I, the latter being detectable only shortly before hatching (Kroemer et al., 1990), predominantly $CD4^+$ (i.e., Class II-restricted) T cells are activated. These $CD4^+$ ("helper") lymphocytes of donor origin express the high affinity IL-2R and probably produce their own IL-2 and proliferate in an autocrine fashion via the IL-2/IL-2R pathway, as do mammalian $CD4^+$ cells of the $T_{H0}$ or $T_{H1}$ phenotype (Smith, 1988). At the same period, $CD8^+$TCR0 cells of the recipient become activated either by the antigenic makeup of the donor-cell population or more likely by the presence of T helper-derived lymphokines. $CD8^+$ cells have been described by Max Cooper's group in the embryonic and adult chicken (Bucy et al., 1989, 1990). No TCR has been found on these cells, which have been interpreted as candidate natural killer cells in this species. Practically all $CD8^+$TCR0 cells in the GVHR spleen display the IL-2R, thus being capable of proliferating in response to endogenous or exogenous IL-2. In support of a central role of IL-2 in the pathogenesis of splenomegaly, local as well as systemic GVHR of mice or humans are suppressed by monoclonal antibodies directed against the IL-2R (Ferrara et al., 1986; Volk et al., 1986; Cavazzana-Calvo et al., 1990). Similarly, local GVHR in the chicken embryo can be prevented by functional IL-2R blockade with INN-CH-16 (Penninger et al., 1989), that is, a murine mAb that neither binds chicken complement nor exerts direct toxic effects (Schauenstein et al., 1988). The unphysiological presence of activated CD4 donor cells and/or activation of recipient lymphocytes in the spleen undergoing GVHR probably also stimulate the nonspecific proliferation of hematolymphopoietic elements. Thus, the proliferation of donor lymphocytes is outnumbered by the expansion and/or recruitment of host cells either of lymphoid (TCR0), prelymphoid or nonlymphoid type. Experiments about local GVHR also detected a predominance of host cells over cells expressing the donor MHC haplotype in chlorioallantoic pocks (Penninger et al., 1989, 1990).

To summarize, the data reported here point to a significant donor as well as host contribution to GVHR in the chicken embryo and reveal the existence of specific and nonspecific components in splenomegaly development. It should indeed be emphasized that about 80% of white blood cells present in the enlarged spleens do not seem to belong to the lymphoid population. The embryo challenged with an IL-2 stimulation appears to respond by amplifying the cell types present at that time, that is, CD8+ cells and cells that are probably hemopoietic precursors. It remains to be investigated whether proliferating cells display the same subpopulation profile, when GVHR occurs in neonatal rather than embryonic hosts or in adult organisms made immunodeficient by artificial means.

### MATERIALS AND METHODS

### Induction and Quantitation of GVHR

Highly inbred chickens homozygous for either the $B^4$ (CC line) or $B^{12}$ (CB line) MHC haplotypes (obtained from the Czechoslovakian Academy of Sciences, Prague, or from the INRA station in Le Magneraud, France) were used in all experiments. $10^6$ peripheral blood lymphocytes (PBL) were isolated from heparinized blood from 3- to 4-week-old donors by differential centrifugation, washed in phosphate-buffered saline (PBS, pH = 7.2), and injected into a chorioallantoic vein of 13-day-old (E13) syngeneic or allogeneic embryos. Necropsy was performed after 5, 6, or 7 more days of incubation (E18 to E20).

### Cytofluorochemical Analysis

After mechanical dissociation of the embryonic spleen through 200-mesh stainless steel sieves, viable cells (initially, >70%) were purified by means of a Ficoll[R] (Pharmacia, Uppsala, Sweden) gradient, washed in PBS supplemented with 10% heat-inactivated bovine serum, and subjected to immunofluorescence analysis. $1 \times 10^6$ cells/ml were incubated in a first step, either with biotinylated $B^4$ or $B^{12}$-specific alloantisera (gift from Dr. D. Bouret) or with the monoclonal antibodies (mAbs) CT4 (specific for CD4, Chan et al., 1988), CT8 (CD8, Chan et al., 1988), TCR1 ($\gamma/\tau$ TCR, Sowder et al., 1988; kindly provided by Dr. Max Cooper), TCR2 ($\alpha/\beta$ TCR, Chen et al., 1988; gift from Dr. Cihak), 319D-3 (specific for B cells; gift from Dr. F. Coudert), or INN-CH-16 (directed against the inducible light chain of the IL-2 receptor, CD25; Hàla et al. (1986; obtained from Dr. Karel Hàla), which were used at optimal concentrations established in preliminary experiments. After three washes (600 ×g, 5 min, 4°C), fluorescence was developed using either streptavidin-phycoerythrin conjugates (Southern Biotechnology, Birmingham, AL), antimouse pan Ig-FITC (Nordic, Tilburg, Netherlands) or phycoerythrin, antimouse IgM texas red (Southern, for INN-CH-16), or antimouse IgG1-FITC (Southern, for all other monoclonal antibodies). All incubations were performed on wet ice for 30 min, followed by three washes. Stained cells were analyzed using automated cytofluorometry (FACS IV, Becton-Dickinson) or examined visually by fluorescence microscopy. For each value presented (mean ± standard error of the mean, SEM) three to eight experimental series (each comprising two to five animals) were performed. Group differences were calculated using the Mann–Whitney test.

### ACKNOWLEDGMENTS

The authors express their gratitude to Drs. Cooper, Chen-Lo-Chen, Cihak, Coudert, Hála, and Bouret for the gift of valuable serological reagents, and to Mrs. A. Lehmann for expert technical assistance.

*(Received November 1, 1990)*

*(Accepted November 21, 1990)*

### REFERENCES

Bril H. and Benner R. (1985). Graft-vs-host reactions: mechanisms and contemporary theories. CRC Critical Reviews in Clinical Laboratory Science. **22:** 43–95.

Bucy R.P., Chen C.H., and Cooper M.D. (1990). Development of cytoplasmic CD3+/T cell receptor-negative cells in the peripheral lymphoid tissue of chickens. Eur. J. Immunol. **20:** 1345–1350.

Bucy R.P., Coltey M., Chen C.H., Char D., LeDouarin N.M., and Cooper M.D. (1989). Cytoplasmic $CD3^+$ surface $CD8^+$ lymphocytes develop as a thymus-independent lineage in chick-quail chimeras. Eur. J. Immunol. **19:** 1449–1455.

Cavazzana-Calvo M., Fromont C., Le Deist F., Lusardi M., Coulombel L., Deroca J.-M., Gerorta I., Griscelli C., and Fischer A. (1990). Specific elimination of alloreactive T cells by and anti-interleukin-2 receptor B chain-specific immunotoxin. Transplantation **50:** 1–7.

Chan M.M., Chen C.H., Ager L.L., and Cooper M.D. (1988). Identification of the avian homologues of mammalian CD4 and CD8 antigens. J. Immunol. **140:** 2133–2138.

Chen C.H., Ager L.L., Gartland G.L., and Cooper M.D. (1986). Identification of a T3/T cell receptor complex in chickens. J. Exp. Med. **164:** 375–380.

Chen C.H., Cihak J., Lösch U., and Cooper M.D. (1988). Differential expression of two T cell receptors, TcR1 and TcR2, on chicken lymphocytes. Eur. J. Immunol. **18:** 539–543.

Désveaux-Chabrol J. and Dieterlen-Lièvre F. (1987). A comparative study of the GVH-R in the chicken embryo: effects of various allogeneic cells and various administration routes. Dev. Comp. Immunol. **11:** 179–190.

Désveaux-Chabrol J., Gendreau M., and Dieterlen-Lièvre F. (1989). Ontogeny of the GVH-R inducing capacity in conventional and germ-free chickens. Dev. Comp. Immunol. **13:** 65–71.

Fedecka-Bruner B., Désveaux-Chabrol J., and Dieterlen-Lièvre F. (1989). T cells in the spleen of chicken embryos undergoing a graft-versus-host reaction. Prog. Clin. Biol. Res. **307:** 19–29.

Ferrara J., Maron A., McIntyre J.F., Murphy G.F., and Burakoff S.J. (1986). Amelioration of acute graft vs host disease by in vivo administration of anti-interleukin 2 receptor antibody. J. Immunology **137:** 1874–1879.

Gleichmann E., Pals S.T., Rolink A.G., Radaszkiewicz T., and Gleichmann H. (1984). Graft-versus-host reactions: clues to the etiopathology of a spectrum of immunological diseases. Immunol. Today **5:** 324–332.

Hàla K., Schauenstein K., Neu N., Kroemer G., Wolf H., Boeck G., and Wick G. (1986). A monoclonal antibody reacting with a membrane determinant expressed on activated chicken T lymphocytes. Eur. J. Immunol. **16:** 1331–1336.

Korngold R. and Sprent J. (1985). Surface markers of T cells causing lethal graft-vs-host disease to class I vs class II H-2 differences. J. Immunol. **135:** 3004–3010.

Kroemer G., Guillemot F., Bernot A., Zoorob R., Park I., Billault A., Béhar G., and Auffray C. (1990). The chicken major histocompatibility complex (MHC): evolutionary conserved class I and class II genes are closely associated with non-MHC genes. *In:* The avian model in developmental biology: from organisms to genes, LeDouarin N. and Dieterlen, F., Eds. (Paris: Editions du CNRS), in press.

Laferty K.J., Walker K.Z., Scollay R.G., and Kilby V.A.A. (1972). Allogeneic interactions provide evidence for a novel class of immunological reactivity. Transplant Rev. **12:** 198–205.

McBride R.A. (1966). Graft-versus-host reaction in lymphoid proliferation. Cancer Res. **26:** 1135–1151.

McBride R.A., Coppleson L.W., Nisbet N.W., Simonsen M., Skowron-Cendrzak A., and Wigzell H.L.R. (1966). Accelerated immunological maturation in the chick. Immunol. **10:** 63–79.

Nisbet N.W., and Simonsen, M. (1967). Primary immune response in grafted cells. J. Exp. Med. **125:** 967–981.

Owen J.J.T., Moore M.A.S., and Harrison G.A. (1965). Chromosome marker studies in the graft-versus-host reaction in the chick embryo. Nature **207:** 313–315.

Parkman, R. (1989). Graft-versus-host disease: an alternative hypothesis. Immunol. Today **10:** 362–364.

Penninger J., Hàla K., and Wick G. (1990). Intra-thymic nurse cell lymphocytes can induce a specific graft-versus-host reaction. J. Exp. Med. **172:** 521–529.

Penninger J., Klima J., Kroemer G., Dietrich H., Hàla K., and Wick G. (1989). Intrathymic nurse cell lymphocytes can induce graft-versus-host reactions with high efficiency. Dev. Comp. Immunol. **13:** 313–327.

Schauenstein K., Kroemer G., Hàla K., Boeck G., and Wick G. (1988). Chicken-activated-T-lymphocyte-antigen (CATLA) recognized by monoclonal antibody INN-CH 16 represents the IL-2 receptor. Dev. Comp. Immunol. **12:** 823–831.

Seto F., and Albright J.F. (1965). An analysis of host and donor contributions to splenic enlargement in chick embryos inoculated with adult chicken spleen cells. Dev. Biol. **11:** 1–24.

Simonsen M. (1957). The impact in the developing embryo and newborn animal of adult homologous cells. Acta Pathol. Microbiol. Scand. **40:** 480–491.

Simonsen M. (1962). Graft versus host reactions. Their natural history and applicability as tools of research. Prog. Allergy **6:** 349–467.

Simonsen M. (1985). Graft versus host reactions: the history that never was, and the way things happened to happen. Immunol. Rev. **88:** 5–23.

Smith K.A. (1988). Interleukin 2: inception, impact, and implications. Science **240:** 1169–1176.

Sowder J.T., Chen C.H., Ager L.L., Chan M.M., and Cooper M.D. (1988). A large subpopulation of avian T cells express a homologue of the mammalian T$\gamma/\delta$ receptor. J. Exp. Med. **167:** 315–322.

Sprent J., Schaefer M., and Korngold M. (1990). Role of T cell subsets in lethal graft-versus-host disease (GVHD) directed to class I versus class II H-2 differences. J. Immunol. **144:** 2946–2954.

Volk H.-D., Brocke S., Osawa H., and Diamantstin T. (1986). Suppression of the local graft-vs.-host reaction in rats by treatment with a monoclonal antibody specific for the interleukin 2 receptor. Eur. J. Immunol. **16:** 1309–1312.

Yassine F., Fedecka-Bruner B., and Dieterlen-Lièvre F. (1989). Ontogeny of the chick embryo spleen—a cytological study. Cell Dif. Dev. **27:** 29–45.

